# Supplementary material for: Exploring pathways to compulsory detention and ways to prevent repeat compulsory detentions in England; clinician perspectives
Source: PLOS Ment Health. 2025 Jun 20;2(6):e0000314. doi: 10.1371/journal.pmen.0000314 (PMC12798629; doi:10.1371/journal.pmen.0000314)
Supplement: Attachment — Submitted filename: Response to Reviewers.docx [file pmen.0000314.s004.docx]

Dear Editorial Team,

Thank you for the opportunity to revise and resubmit our manuscript titled "Exploring Pathways to Compulsory Detention and Ways to Prevent Repeat Compulsory Detentions: Clinician Perspectives," for consideration for publication in *PLOS Mental Health.*

We greatly appreciate the time and effort that you and the reviewers have invested in providing constructive feedback. We have carefully considered all the comments and have revised address each point raised. Below, we provide a detailed response to the reviewers’ comments on pages 2-5.

Yours Sincerely,

**LONDON’S GLOBAL UNIVERSITY**

Dr Ariana Kular

**E:** [a.kular@ucl.ac.uk](mailto:a.kular@ucl.ac.uk)

| **Reviewer Comments** | **Response to Reviewers** |
| --- | --- |
| Provide Consolidated criteria for reporting qualitative research (COREQ) checklist  While the manuscript aligns well with COREQ guidelines in many areas, the following aspects could be better addressed to fully meet the standards:  -Include a statement about the researchers’ backgrounds, biases, and relationship with participants.  -Explicitly describe the reflexivity process undertaken during data collection and analysis.  -Provide more details on achieving data saturation.  -The manuscript generally demonstrates good adherence to EQUATOR guidelines via COREQ but would benefit from addressing the gaps noted above. | This has been completed and uploaded as a separate document in the submission platform.  We have added additional information about the research team. A statement regarding relationship with participants was added in the data collection section.  We have added information about reflexivity in the last paragraph of the research team section. Reflexivity undertaken during data analysis is also described in the data analysis section.  We did not aim for data saturation, as this approach is not aligned with Braun and Clarke's framework for analysis. Instead, our goal was to interview staff from a diverse range of backgrounds, and recruitment ceased once we had achieved a broad representation of participants.  Thank you, the above comments have been addressed. |
| Abstract: The term, "template analysis" is used without explanation, which may be unclear to some readers. Brief clarification might enhance accessibility for a broader audience. | A short explanation has now been added following the mention of template analysis.  *“Data were analysed using template analysis, which involved developing a structured framework to organise and analyse data based on key themes.”* |
| Abstract: The abstract does not mention any limitations of the study, which are often an essential part of understanding the scope and applicability of the findings. | In the conclusion section of the abstract, we have summarised the limitations of our study. |
| Abstract: While the abstract mentions findings and suggestions, it could be more explicit about how these findings contribute new knowledge to the field or how they advance existing literature or practice guidelines. | We have adapted the conclusion of the abstract to reflect how our study contributes to new knowledge and practice, within the scope of the word limit for the abstract. |
| Methods – Research Team: Authors may want to define the term gender for clear explanation in the methodology. They should be clear whether they are implying to “gender” (social construct and how individuals identify themselves?) or “sex”( identity assigned at birth) to be more specific. | This has clarified within the research team section. |
| Methods – Participants: Considering the study's reliance on clinician perspectives, there is a risk of interpretation bias. Therefore, this point may be higgled in the limitations. | This has been added to the limitations section of the discussion. |
| Methods – Participants: While the paper focuses effectively on clinician perspectives, it misses the opportunity to integrate service user insights directly into this study. Including these perspectives could provide a more balanced view of the factors leading to compulsory detention. This point may also be added to the study limitations stating the scope of the study was limited to clinician perspectives only.  Results – Thematic Analysis: The paper relies heavily on qualitative data from clinician interviews. While this provides depth, supplementing these insights with quantitative data or existing empirical studies would have strengthened the arguments and would have provided a more robust evidence base. Therefore, lack of “data triangulation” using other assessment approaches should be mentioned as the study limitation. | Thank you for your comment. Our team has submitted a companion paper to the PLOS Mental Health journal, which explores the same topic from a service user perspective. This have been reviewed by your journal team and is currently has minor revisions to be made. It can be found here: <https://doi.org/10.1101/2024.06.04.24308425>  This has been added to the limitations section of the discussion. |
| Results – Thematic Analysis: Themes like medication non-adherence or cultural attitudes might be oversimplified without delving deeper into the complexities of individual cases  For example: A deeper analysis would consider reasons for non-adherence, such as side effects that outweigh perceived benefits, lack of understanding about the medication, cultural or personal beliefs that discourage medication use, or practical issues like cost or difficulty accessing pharmacy services. Similarly, A more thorough examination would look at historical mistrust of healthcare systems, language barriers, cultural stigma around mental illness, or differing cultural interpretations of mental health symptoms. | Thank you for your comment. Upon reviewing all the quotes under the theme of medication non-adherence, it became apparent that most clinicians did not explore in detail why patients stopped taking their medication, and did not delve into individual cases, often describing medication non-compliance as a commonly observed phenomenon. Among the few clinicians who did offer brief insights, the three reasons cited included patients believing they had recovered, medication being prescribed at a dose too low to impact their mental state, and a general distrust of psychiatric medication. This additional information has now been added, with one additional supporting quote within the manuscript, and one within the supplementary table of quotes.  Additionally, upon reviewing the quotes for the cultural attitudes theme, only a few clinicians briefly addressed the cultural attitudes towards mental illness and mental health services held by certain ethnic communities. One clinician [P9] discussed this topic most extensively. We have incorporated additional insights from the participants' quotes into the manuscript and replaced some of the original quotes in the supplementary table to reflect this. Historical mistrust is currently addressed in the subtheme, which has a revised title, ‘Historical Lack of Trust’, previously entitled ‘Previous failure to build trust’. |
| Results – Thematic Analysis: *[Regarding sub-theme, clinician biases and assumptions]* While clinician biases, especially racial biases, are crucial issues, the paper could benefit from exploring this theme in a more balanced way, alongside other systemic and structural factors influencing detention practices.  For example: a mental health service where clinician bias is reported as a key issue leading to higher detention rates among Black men. This is an important observation. However, alongside clinician biases some other factors such as resource limitations, insufficient community support services available due to funding cuts, health policy/law influences and training and support of healthcare providers should also be explored.  Results – Thematic Analysis: *[regarding sub-theme - Systemic and Institutional Barriers to Engagement]* Although the paper suggests some systemic solutions, it could offer more detailed strategies for implementing these solutions, such as specific policy changes or training programs that could effectively address identified issues. | Thank you for your comment. Our theme, *‘Service/Clinician Level Factors Contributing to People Being Compulsorily Detained,’* does include sub-themes addressing systemic and structural factors such as poor communication and continuity of care, limited resources, disruptions during care, lack of treatment variety, and other institutional barriers that hinder engagement and may contribute to the likelihood of compulsory detention. We have reworded the opening sentence for the overall theme to highlight that these issues will be discussed in the results.  Upon reviewing all quotes, while participants provided a range of suggestions for service improvements—such as better resourcing of community services, avoiding overly rapid hospital discharges, and fostering collaborative care planning—they did not offer specific, detailed strategies for implementing these changes. These service change suggestions have been outlined in theme three *“potential pathways to reducing compulsory detention.”* |
| Discussion: Discussion section, while comprehensive, could be more succinctly presented. Clearly relating each point to the results of the study will help reinforce the study's contributions and highlights how the findings support the broader narrative or implications being discussed.  For example, if the study found a statistical correlation between certain systemic factors and detention rates, ensure this is explicitly stated and discussed in the context of existing research or theory  It would be more beneficial to clearly highlight specific biases identified in the study, instead of broadly discussing clinician biases, and discuss their observed impact on detention rates, directly referencing study findings | We have made our statement of principal findings more concise and amended the discussion of these findings in relation to existing literature. First, patient-level factors are addressed, followed by service-level, clinician-level factors, and potential strategies for reducing compulsory detention, with each section contextualised by the existing literature.  As a qualitative study, no statistical correlations were identified; however, we have explored the role of systemic factors in detention, drawing on relevant research.  The biases and assumptions discussed by clinicians, including those related to prior psychosis diagnosis, age, gender, and race, are examined in detail within the discussion, with a focus on their impact as reflected in existing literature. The qualitative nature of the study precluded very specific analysis of associations between biases and impacts on detention rates. |
| Discussion – Limitations of Study: As correctly mentioned in the limitations, not only the sample is predominantly composed of psychiatrists, but authors may also want to emphasize that sample was drawn extensively from London, which may limit the generalizability of the findings. It is therefore suggested to include the geographical location in the “title”, so as to give the readers the full scope of the study setting and results. | Thank you for your comment. As not all participants were based in London, we would prefer the title to remain more general and not reference a specific geographical location, but we have added ‘in England’ to the title. |
